# Effect of overload on changes in mechanical and structural properties of the annulus fibrosus of the intervertebral disc

**DOI:** 10.1007/s10237-021-01505-w

**Published:** 2021-08-24

**Authors:** Małgorzata Żak, Celina Pezowicz

**Affiliations:** Department of Mechanics, Materials and Biomedical Engineering, Faculty of Mechanical Engineering, Wrocław University of Science and Technology, Łukasiewicza 7/9, 50-371 Wrocław, Poland

**Keywords:** Intervertebral disc, Annulus fibrosus, Three-joint complex loading, Pathological model, Mechanical properties

## Abstract

The research focussed on analysing structural and mechanical properties in the intervertebral disc (IVD), caused by long-term cyclic loading. Spinal motion segments were divided into two groups: the control (C), and the group in which it was analysed the impact of posterior column in the load-bearing system of the spine—specimens with intact posterior column (IPC) and without posterior column (WPC). To evaluate the structural and mechanical changes, the specimens were tested with simulation of 100,000 compression-flexion load cycles after which it was performed macroscopic analysis. Mechanical properties of the annulus fibrosis (AF) from the anterior and posterior regions of the IVD were tested at the uniaxial tension test. The stiffness coefficient values were statistically 32% higher in the WPC group (110 N/mm) than in the IPC (79 N/mm). The dynamics of increase in this parameter does not correspond with the course of decrease in height loss. WPC segments revealed clear structural changes that mainly involve the posterior regions of the IVD (bulging and delamination with the effect of separation of collagen fibre bundles). Pathological changes also caused decreases in the value of stress in the AF. The greatest changes in the stress value about group C (7.43 ± 4.49 MPa) were observed in the front part of the fibrous ring, where this value was for IPC 4.49 ± 4.78 MPa and WPC 2.56 ± 1.01 MPa. The research indicates that the applied load model allows simulating damage that occurs in pathological IVD. And the posterior column’s presence affects this change’s dynamics, structural and mechanical properties of AF.

## Introduction

In the spinal motion segment, also known as the functional spinal unit (FSU), load transfer is spread over a three-point complex: the intervertebral disc (IVD) and two articular processes. Studies have so far indicated that the compression force resulting from the loads acting on the spine is transferred mostly by the disc and, to a lesser extent, by articular processes (Nachemson 1960; Pezowicz 2017). At the same time, the percentage share of the processes depends on body posture and motor activity (Gellhorn et al. 2013; Iorio et al. 2016). Many experimental in vitro studies (Dunlop et al. 1984; Yang et al. 1984; Skipor et al. 1985) confirm these observations, emphasising that the action of additional moments of forces in the vertical axis and in the sagittal plane causes a significant increase in the load on the articular processes. Adams et al. (Adams et al. 1980) showed that in a system stimulating prolonged (approx. 3 h) standing in an upright position, 16% of the load acting in the lumbar spine is indeed transferred through articular processes, while during short-term standing (approx. 5 min) it corresponds to just 4%.

Under the actions of the complex load system (axial compression with extension or flexion in the range of 4°–6°), articular processes transfer from 10 to 40% of the applied load (Dunlop et al. 1984; Rousseau et al. 2006; Schulz et al. 1991). At the same time, the increase in the force acting on the articular processes during extension and flexion increases progressively with time (Adams et al. 1980; Dunlop et al. 1984; Shirazi-Adl et al. 1987) At the same time, it shows the importance of the spinal loading implementation scheme on changes in the distribution of forces in the anterior and posterior columns.

The relationship between support points in the anterior column (the IVD) and the posterior column (articular processes) is a kind of kinematic chain, in which degeneration or injury of one link increases the likelihood of degenerative changes in another one. Moreover, changes in the biomechanics of one of the FSUs affects the mechanics of adjacent elements, causing multi-level degeneration of the spine (Iorio et al. 2016) primarily initiated by structural changes within IVD.

The IVD is characterised by a composite structure, in which collagen fibres form the matrix and proteoglycans and non-collagenous proteins are the binding material. Owing to this structure, the material exhibits high mechanical strength. However, the anisotropic properties and the complex loading conditions acting on the IVD promote the occurrence of specific damage mechanisms leading to a degeneration of its structures. The still poorly understood pathomechanism of degenerative processes of the IVD indicates that it is not associated exclusively with mechanical injuries resulting from a sudden, dynamic overload of the spine but also with biochemical processes related to hydration in the IVD (Wang et al. 2011).

Spinal degeneration is one of the most frequently mentioned lifestyle diseases, and over 80% of those are in degeneration with the IVD (Adams et al. 2012; Urban et al. 2003). The problem of degeneration and the subsequent changes in the mechanical and structural properties of the IVD is a fundamental issue from the standpoint of the pathomechanism of formation of spinal injuries. Among the large group of factors contributing to the formation of pathological changes, the greatest impact comes from long-term cyclic loading (Johannessen et al. 2004, Hasegawa et al. 1995; Koeller et al. 1984, Schollum et al. 2018) leading to the development of fatigue processes of the disc.

It should be noted, however, that the results of many conducted studies do not provide a full assessment of the impact of loads, especially of a cyclic nature on the process of degenerative changes within the IVD. At the same time, more and more attention is paid to the influence of biomechanics and physiology of the facet joints and their relationship to the development of IVD degeneration (Szkoda-Poliszuk et al. 2021).

Therefore, our study aimed to analyse the overload changes occurring in the IVD caused by long-term cyclic loading. The research concerned in particular analysis of the impact of degenerative and disease changes posterior column of the spine (articular processes) on changes of overload in the IVD. In the adopted animal model (pig model), this process was induced by removing the posterior column. The impact of cyclic loads on the IVD was performed based on mechanical properties of the annulus fibrosus (AF) determined in the uniaxial tensile test.

## Material and methods

The study was conducted on an animal model consisting of 10 lumbar spine regions collected *post-mortem* from domestic piges (aged: 8–9 months, weight: 90–120 kg). The following FSUs were isolated from eight lumbar regions: L2–L3, L3–L4, and L4–L5. The segments were cleaned of soft tissue, leaving intact junctions between vertebral bodies and the IVD and within the zygapophysial joints. In total, 14 FSUs were obtained, which were subjected to cyclic compression loading. Six segments from the other two lumbar regions constituted the control group (C) as physiological specimens with the unchanged structure of the IVD. All prepared specimens were stored in separate plastic bags at a temperature of − 20 °C until testing.

The study protocol consisted of three steps:

1. Assessment of the impact of high-cycle complex loading conditions both in segments with the complete articular triad and in segments with the posterior column (articular processes) removed,
2. Analysis of the mechanical properties of the AF (in the uniaxial tension test) depending on the location (anterior/posterior IVD),
3. Macroscopic structural analysis of the IVD.

### Cyclic compression-flexion test protocol

The specimens were thawed for 5 h at room temperature and then hydrated for one hour in normal saline. Due to the major role of the articular triad in the transfer of loads affecting the spine, 14 FSUs were divided into two experimental groups. The first group (*n* = 7) consisted of segments with intact posterior column (IPC). By contrast, in the second group (*n* = 7), the articular processes were removed to obtain segments without posterior column (WPC) but with intact anterior column (junction between the vertebral bodies and the IVD).

Cyclic tests of FSUs have carried out a simulation of 100 000 axial load cycles in the range of 150–650 N with a frequency of 2 Hz (an MTS-858-Mini-Bionix testing machine) (Żak 2014).

Simultaneously with compression load, physiological bending of the segment was forced at an angle of 6°. During the test, constant hydration was ensured by supplying normal saline through the superior vertebral body and wrapping with moist gauze eliminating, assuming that the predominant direction flows through the marrow contact channels in the endplate (Żak 2014; Ayotte et al. 2001). The obtained force–displacement characteristics were used to estimate the change in the height of the IVD (Δ*h*) and the stiffness coefficient (*k*) in the load range of 630–650 N.

### Macroscopic analysis protocol

An assessment of structural changes in the IVD was carried out after the completion of the cyclic loading test and involved two undamaged (IPC) and two damaged (WPC) FSUs. The segments were fixed in 7% glutaraldehyde solution for seven days. In addition, one segment from the C group was fixed, which was comparative material with unchanged structure. Using a Struers Accutom-5 precision cutter, the segment was cut into 2 mm thick slices in the sagittal plane. A macroscopic image of the examined structures was recorded using a Zeiss SteREO Discovery.V20 stereomicroscope.

### Uniaxial tension test protocol

Uniaxial tension testing was carried out for a group of 10 FSUs that were previously subjected to cyclic loading (5—IPC and 5—WPC) and 5 segments constituting the C group. An IVD together with adjacent halves of the vertebral bodies (VB–IVD–VB) were isolated from each segment. Specimens containing the outer AF together with a fragment of the vertebral body were excised in the frontal plane from the anterior (A) and posterior (P) regions of the IVD, which were then divided symmetrically into two parts (halves) (Żak et al. 2013). A total of 60 multi-lamellae annulus fibrosus specimens (20 in the IPC group, 20 in the WPC group and 20 in the C group) were obtained—with 10 samples in each group from the anterior and 10 samples from the posterior.

Before testing, each specimen was subjected to 30-min hydration in 0.15% normal saline solution at room temperature (Żak et al. 2016). The dimensions of the geometrical parameters of the multilayer annulus fibrosus specimens in each group are presented in Table 1.

**Table 1:** Summary of average geometric dimensions of the specimens with bone attachment from the three study groups: the control group (C), the group with intact posterior column (IPC) and the group without posterior column (WPC) after 30-minute hydration in 0.15% normal saline solution at room temperature

Study groups	Location of the annulus fibrosus	L [mm]	W [mm]	T [mm]	Multilayer annulus fibrosus specimen
C	A (*n* = 10)	6.24 ± 0.95	15.50 ± 1.68	4.00 ± 0.48	
	P (*n* = 10)	5.96 ± 1.16	16.00 ± 1.48	4.00 ± 0.64	
IPC	A (*n* = 10)	5.26 ± 0.27	14.68 ± 0.69	3.75 ± 0.32	
	P (*n* = 10)	3.94 ± 0.73	13.73 ± 0.93	3.40 ± 0.35	
WPC	A (*n* = 10)	4.80 ± 0.24	14.60 ± 0.64	3.17 ± 0.49	
	P (*n* = 10)	4.60 ± 0.57	13.64 ± 1.21	3.02 ± 0.47	

Average dimensions of the specimens: L—Length, W—Width, T—Thickness, Location of the annulus fibrosus: A—Anterior, P—Posterior, n—Number of samples

An MTS Synergie 100 testing machine carried out the uniaxial tensile test at a crosshead speed of 0.5 mm/s until specimen failure (with five pre-conditioning cycles by applying a 1 mm displacement).

Based on the obtained force–displacement and stress–strain characteristics, selected mechanical parameters were determined, stiffness coefficient (*k*), ultimate tensile strength (*σ*_UTS_), strain to failure (*ε*_N_), and Young’s modulus (*E*). The values of *k* and *E* were determined in the linear range of characteristics. The results were presented as means with standard deviation. Statistical analysis was performed using the Origin-7 program. The normality of distribution of the results was analysed using the Shapiro–Wilk test, which can also be used to analyse small samples. The results were presented as means with standard deviation. On the other hand, in order to compare the means between the individual study groups, ANOVA was used with a significance level of 0.05.

## Results

### Cyclic compression-flexion test

**Fig. 1:**
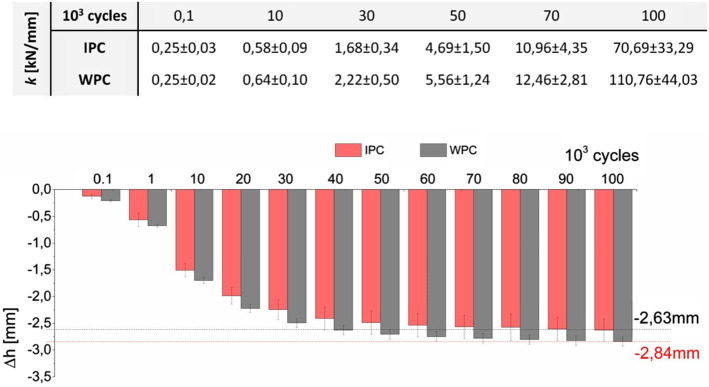
The characteristics of a decrease in the intervertebral disc (IVD) height (Δ*h*) in undamaged (IPC) and damaged (WPC) segments, with indication of the maximum height decrease and change in stiffness (*k*) over subsequent load cycles

The dynamics of change in the IVD height (Δ*h*—disc height loss) shows two distinctive ranges. In the initial phase, there is a rapid decrease in height loss, which occurs during the first 40,000 cycles in the undamaged IPC segments. By contrast, damaged WPC segments show a progressive height loss decrease that persists until 60,000 load cycles (Fig. 1). Later in the Δ*h* graph, the height fluctuations are insignificant and stay at a similar level (2.63 ± 0.21 mm for IPC and 2.84 ± 0.09 mm for WPC).

The stiffness coefficient (*k*) of the tested segments increases with successive load cycles, but the dynamics of increase in this parameter do not correspond with decrease in height loss—Fig. 1. In the undamaged IPC segments, the initial value *k* was 0.25 ± 0.03 N/mm and was similar to the value in the damaged WPC segments of 0.25 ± 0.02 kN/mm. The posterior column removal effect was visible only after 50,000 cycles when the coefficient *k* in the damaged WPC segments amounted to 5.56 ± 1.24 kN/mm and was higher than in undamaged IPC segments (4.69 ± 1.50 kN/mm). After 100,000 cycles, the highest stiffness coefficient was observed in the WPC segments (110.76 ± 44.03 kN/mm), which was higher by 32% than in the IPC segments (76.69 ± 33.29 kN/mm).

### Uniaxial tension test

**Fig. 2:**
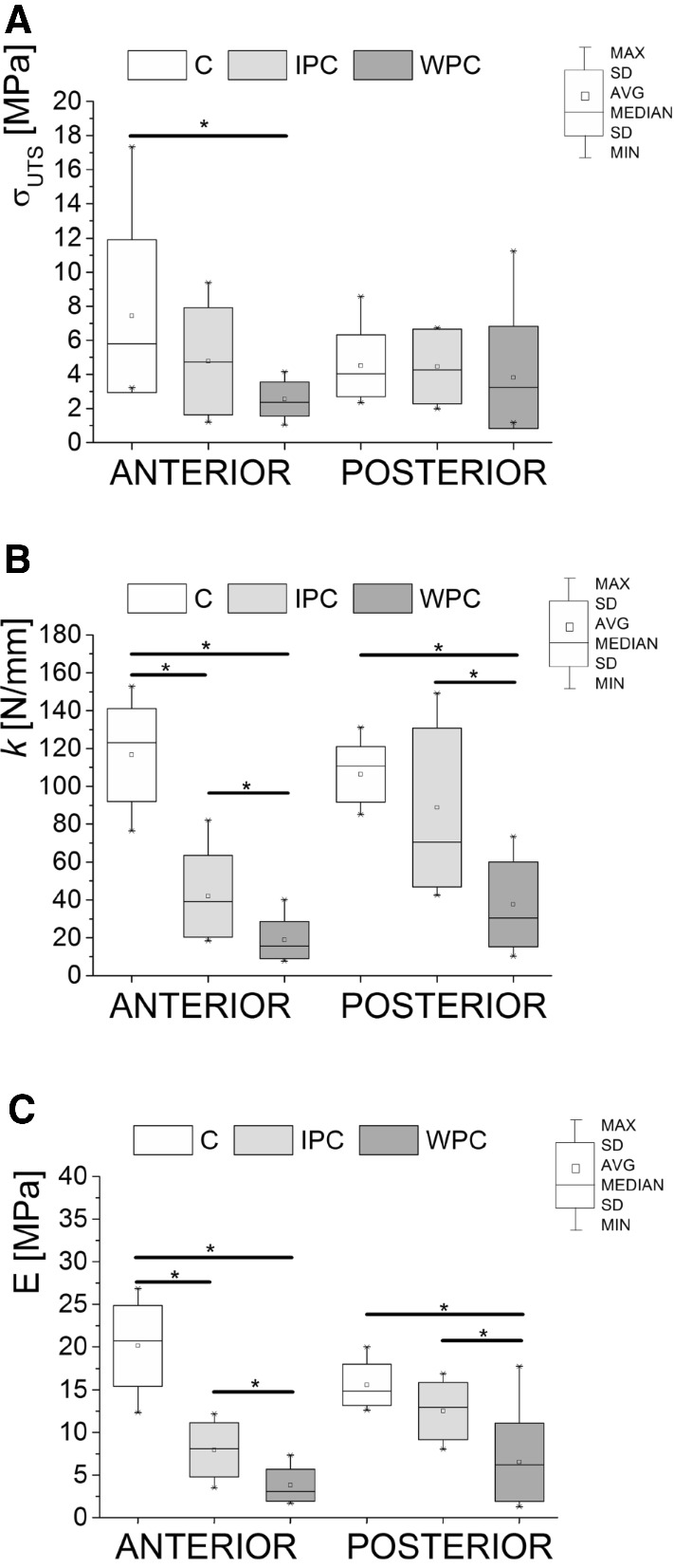
Mechanical properties in the C group (*n* = 10) and the pathological groups: IPC with processes and WPC without processes (*n* = 10) of the annulus fibrosus (AF) from the anterior (A) and posterior (P) IVD; **a** ultimate tensile strength (*σ*_UTS_), **b** stiffness (*k*), and **c** Young’s modulus (*E*). *Statistical significance (*p* < 0.05)

The value of the ultimate tensile strength (*σ*_UTS_) of the AF in pathological specimens (IPC and WPC groups) was smaller than in the controls, with statistically significant differences demonstrated only for the anterior IVD (Fig. 2a). In the anterior IVD, the recorded *σ*_UTS_ was one and a half times smaller in the case of the IPC group (4.49 ± 4.78 MPa) and three times smaller in the case of the WPC group (2.56 ± 1.01 MPa) compared to the C group (7.43 ± 4.49 MPa). In the posterior IVD, the ultimate tensile strength was 4.52 ± 1.82 MPa in the C group, 4.47 ± 2.19 MPa in the IPC group, and 3.83 ± 3.00 MPa in the WPC group.

Long-term cyclic loading had a statistically significant effect (*p* < 0.05) on decreases in the stiffness coefficient (*k*) and Young’s modulus in pathological specimens (Fig. 2b, c). In the anterior IVD, there was a nearly three-fold decrease in the stiffness coefficient in the IPC group (41.87 ± 21.57 N/mm) and a more than six-fold decrease in the *k* value in the WPC group (18.85 ± 9.78 N/mm) compared to the group of specimens in the control state (116.62 ± 24.57 N/mm). On the other hand, Young’s modulus was more than twice lower in the IPC group (7.95 ± 3.18 MPa) and as much as five times lower in the WPC group (3.82 ± 1.86 MPa) compared to the C group (20.13 ± 4.72 MPa).

In the posterior IVD, the C group’s *k* value was 106.39 ± 14.64 N/mm, whereas Young’s modulus stayed at 15.59 ± 2.43 MPa. In the IPC group, the *k* value decreased to 88.81 ± 41.99 N/mm and Young’s modulus decreased to 12.52 ± 3.37 MPa. In the WPC group, the *k* value was 37.68 ± 22.41 N/mm, while the E value was 6.52 ± 4.58 MPa; these values were statistically lower than those obtained in the previous two groups (C and IPC).

### Macroscopic analysis

**Fig. 3:**
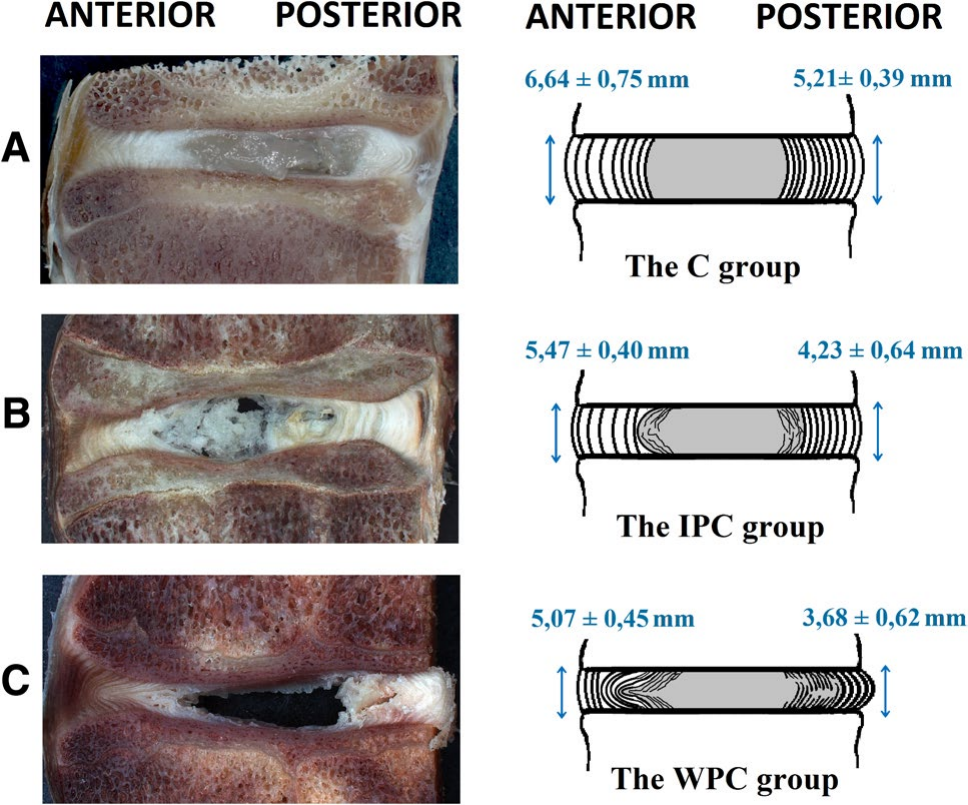
Macroscopic images of sagittal cross sections through glutaraldehyde-fixed FSUs with the change in the height of the interbody space in the anterior and posterior regions of the IVD: **a** the control group (C), **b** the group with intact posterior column (IPC), **c** the group without posterior column (WPC)

Cross sections of physiological and pathological segments revealed no changes in the vertebral bodies and endplates. By contrast, clear structural changes appeared within the IVD (Fig. 3). The complex loading conditions used during cycling testing of the segments contributed to structural changes, particularly within the AF’s lamellae in the posterior and posterolateral IVD. The main changes concerned penetration of the nucleus pulposus (NP) through the AF’s inner lamellae and heterogeneity in adjacent lamellae caused by delamination (Fig. 3). In the IPC segments, the area of pathological changes is limited to the inner structures of the AF (Fig. 3b). By contrast, in the WPC segments, this area is much larger and mainly involves the posterior IVD (Fig. 3c).

Additionally, in the sagittal planes were estimate the change in the height of the IVD in the anterior (A) and posterior (P) regions of the IVD. In the case of the control group (not subjected to cyclical loads), the average value of the height of the IVD was 6.64 ± 0.65 mm in the anterior and 5.21 ± 0.39 mm in the posterior (P) regions of the IVD. The values obtained in the groups after the fatigue tests were smaller lower than those in the control group, with a greater decrease was recorded for the posterior IVD. In the anterior IVD, the IPC group’s height of the IVD value was 5.47 ± 0.40 mm, whereas the WPC group’s decreased to 5.07 ± 0.45 mm. While in the posterior IVD, the IPC group’s height of the IVD value was 4.23 ± 0.64 mm, whereas the WPC group’s decreased to 3.68 ± 0.62 mm.

**Fig. 4:**
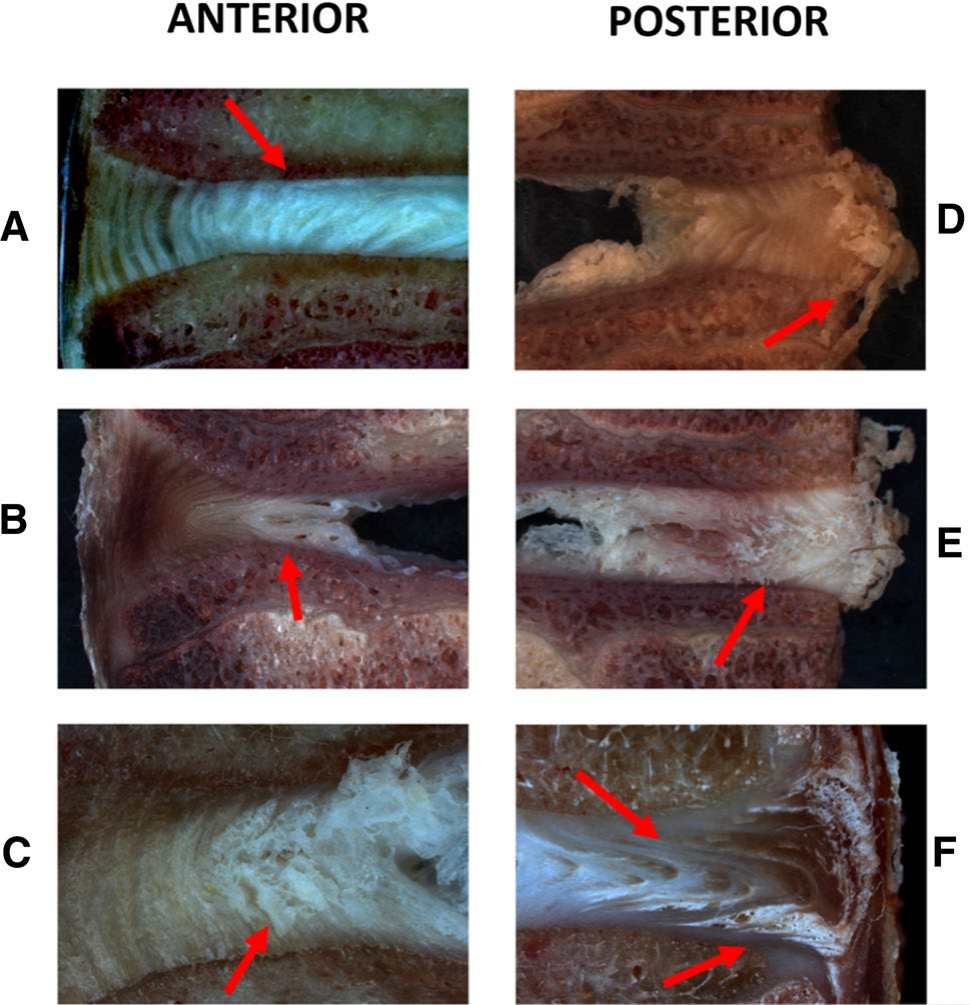
An example of changes in the AF layers in the anterior and posterior IVD isolated from the group without posterior column (WPC): **a** separation of the inner lamellae at the border with the endplate, **b** bulging of the inner layers outwards, **c** damage to the AF lamellae, **d** bulging of the IVD, **e** bulging of the outer layers and damage of the inner layers, **f** delamination with the effect of separation of collagen fibre bundles at the border of adjacent lamellae of the AF with bulging towards the inferior borders of the IVD

As a result of the applied complex loading, the NP in the WPC segments was displaced towards the posterior IVD, causing it to bulge. Besides, the decrease in height (Δ*h*) of the IVD caused a significant progression of structural changes in the AF in the form of outward bulging of the inner lamellae (located near the NP). In the WPC segments, delamination also occurred in the anterior AF in the form of disorganised lamellar distribution and separation of the inner lamellae from the superior endplate (Fig. 4a, b). The bulging in the posterior IVD may take the form of bulging of the outermost layers of the AF (Fig. 4d, e) or proceed with a visible bulge shift towards the inferior border of the IVD (Fig. 4f). The bulging of the AF in the posterior IVD also resulted in the loss of cohesion between adjacent inner and outer lamellae (Fig. 4e). Propagation of bulging also encouraged delamination in the AF with the effect of separation of the fibre bundles at the adjacent lamellae border (Fig. 4e, f).

## Discussion

Lesions of the lumbar spine are strongly correlated with radial damage of the AF progressing towards the periphery of the IVD (Smith et al. 2009). This creates conditions for penetration of the NP material or ingrowth of nerves (which innervate the AF usually up to 3 mm deep around the circumference) (Stefanakis et al. 2012) along radial fissures. The results presented in this study indicate that the applied load model allows simulating damage and structural changes in the AF that occur in patients with pathological changes in the IVD, including radial fissures, delamination, and displacement of the NP material, which lead to extensive internal disorganisation of the AFs and loss of height of the IVD.

The most common mechanical reason for the formation of pathological changes in the IVD is the action of high-cyclic loads and/or overloads of a complex nature, such as compressive loading with simultaneous bending, usually of low amplitude (Hasegawa et al. 1995; Johannessen et al. 2004; Liu et al. 1983). Research conducted by Johannessen et al. (Johannessen et al. 2004) shows that cycling loading with values similar to physiological loads changes the height and stiffness of the IVD, with these changes being reversible during its relaxation (rest) provided adequate hydration is ensured.

The presented test results show a significant decrease in the height of the IVD after prolonged cyclic loading; however, they also indicate that the posterior column does not significantly influence the change in this value in the form of support on articular processes. Nevertheless, it should be emphasised that the posterior column’s presence affects the dynamics of this change. This is evidenced by a clear difference in the speed at which the decrease in the IVD height (Δ*h*) stabilises, which occurs at 35,000 cycles for the IPC segments and as high as 60,000 cycles for the WPC segments. This difference may be important for the formation of changes in the AF structure, especially in the case of the WPC system, which, as an unstable system or in an unstable equilibrium, is extremely sensitive to overloads (such as the lifting of heavy objects).

The obtained change in height does not result from the geometric dimensions (or the surface-to-height ratio) of the IVD itself in the lumbar region. Both the disc’s surface and height in this spinal region are greater than in the cervical or thoracic regions (Belavy et al. 2018). However, the thoracic region, the IVD height changes are similar and do not exceed 2.48 ± 0.47 mm for IPC and 2.87 ± 0.38 mm for WPC after 100,000 load cycles (Żak 2014). In the thoracic region, the decrease in height stabilises in both experimental groups already after 10,000 cycles (Żak 2014) in contrast to the results obtained for the lumbar region.

However, it should be noted that the value of stiffness (which significantly increases only after 50,000 cycles) indicates a change in the viscoelastic properties of the IVD, associated with a pronounced loss of hydration and the effect of the absence of the posterior column. Therefore, a change in the FSU stiffness resulting from cyclic loading has a much greater biomechanical significance as it determines the AF behaviour depending not only on the height of the disc itself but also on such factors as changes in hydration, viscoelastic properties (O’Connell et al. 2011a, b; Hasegawa et al. 1995; O’Connell et al. 2011a, b), or the hydrostatic pressure exerted by the NP (Wilke et al. 1999; Nachemson et al. 1963). The NP transfers loads radially to the AF. In a healthy IVD, this pressure does not act directly on the entire AF; instead, it indirectly increases tension of collagen fibres in successive layers of the AF. Experimental models show that damage in the IVD propagates from the NP towards the AF, resulting in internal protrusions and radial and circumferential separation of the AF layers of collagen fibres referred to as delamination (Vernon-Roberts et al. 1997).

The complex loading conditions cause compression and stretching of individual areas of the AF with a simultaneous increase in hydrostatic pressure within the NP (Wilke et al. 1999; Nachemson et al. 1963). During compression, the stretching of collagen fibres in the outer layer of the AF causes bulging of the IVD in the radial direction by approx. 0.4–1.0 mm (Szkoda-Poliszuk et al. 2018; Stokes 1987). This is accompanied by the formation of microcracks in the AF structure, whose accumulation leads to fatigue failure and, consequently, to the formation of small radial fissures running from the NP towards the outer layers of the AF.

According to Adams and Dolan (Adams et al. 2012), degenerative changes of the IVD can be determined by damage to the endplate of the vertebral body or damage to the AF itself. Damage to the endplate (such as Schmorl’s nodes) is relatively rare in the lower lumbar region, and damage occurring at the upper two lumbar levels (L1–L2 and L2–L3) is associated with severe degeneration of the IVD. On the other hand, a clear loss of the IVD height is characteristic of the changes caused by the AF pathology (Weiler et al. 2012), and degenerated discs show structural changes in the form of radial fissures, which originate from the NP and propagate in the posterior or posterolateral direction of the IVD. The same structural changes of the IVD were obtained using the proposed protocol of complex cyclic loading. Moreover, the system’s tests with the damaged posterior column allowed to obtain more extensive structural changes, including the anterior AF, which are observed in the case of profound degenerative changes of the IVD. The induced changes in the architecture of the annulus fibrosus are reflected in significantly lower mechanical parameters obtained during uniaxial tension testing. There is one more damage mechanism that should be mentioned here, which occurs at the border between the endplate and the AF and is one of the most common causes of surgical treatment of the IVD (Rajasekaran et al. 2013) and, as suggested by experimental evidence, results from increased shear stresses at the annulus–endplate junction (Veres et al. 2010).

The AF exhibits high stiffness and tensile strength (Wu et al. 1976; Acaroglu et al. 1995; Elliott et al. 2001; Wagner et al. 2004; Guerin et al. 2007) as well as anisotropic and nonlinear behaviour resulting from the distribution of collagen fibres in its structure (Viidik 1973; Hansen et al. 2002; Franchi et al. 2007).

In our research, determining the effect of the resulting pathological changes on the mechanical parameters of the outer layers of the AF is essential for characterising the mechanism of damage in the IVD.

So far, the literature has not shown significant differences in Young’s modulus with the appearance of degenerative changes of the AF. Acaroglu et al. (Acaroglu et al. 1995) indicate that the mean Young’s modulus for the outer layers of the AF from a ‘normal’ IVD is 28 MPa in the anterior part (in our tests: 20.13 ± 4.72 MPa) and 13 MPa in the posterior part (in our tests: 15.59 ± 2.43 MPa). These values decrease as degeneration increases and in the case of severe degeneration in the WPC segments they amount to E = approx. 20 MPa in the anterior part (for IPC in our tests: 3.82 ± 1.86 MPa) and E = approx. 8 MPa in the posterior part (for IPC in our tests: 6.52 ± 4.58 MPa). Guerin also showed no significant differences in Young’s modulus but a strong correlation between the mechanical parameters and collagen fibres’ orientation (predominantly the angle of inclination) with the appearance of degenerative changes (Guerin et al. 2006).

On the other hand, our analyses’ results show statistically significant differences between the physiological disc (C) and pathological discs (relating to both IPC and WPC for *p* = 0.05) in the anterior AF. In the posterior AF, statistically significant differences at *p* = 0.05 were observed only between the control and WPC groups.

The structural analysis of the IVDs subjected to complex cyclic loading in this study indicates that the induced pathological changes were characteristic of degenerative changes of the IVD. As shown by Guerin, degeneration of the IVD significantly affects the ability of collagen fibres to reorient themselves, mainly in terms of reduction of the angle of inclination in response to the axial force, which is reflected in lower mechanical properties obtained in the uniaxial tension test (Guerin et al. 2006).

Investigation and understanding of the processes forming of degenerative changes in the IVD will allow to better determine the direction and procedure for clinical treatment of patients with early diagnosis of spinal pain conditions. Our findings provide some guidelines for conducting experimental research on degenerative changes of the IVD and for interpreting structural changes of the IVD based on magnetic resonance imaging. The obtained results may be useful in selecting boundary conditions and validating numerical models of the IVD.

The work has a limitation resulting mainly from using an animal model in studies on overload changes in the spine, especially in the IVD. Most of the research carried out on dissection preparations uses material that is already characterised by specific degenerative changes (2nd or 3rd grade), which at the outset significantly affects the results obtained. Hence, the adoption of homogeneous research material made it possible to get results based on which broader conclusions can be drawn. Also, the application of highly cyclical compressive loads simulating the spine’s long-term activity is not fully reflected in the real state. In everyday life activity, even long periods of walking are interspersed with rest, which is a relaxation period for the IVD. However, only this type of loading scheme made it possible to obtain structural changes in the AF quickly. One crucial element used in this study to limit disc hydration changes was to ensure a continuous saline supply.

The removal of articular processes to simulate damage to the spine’s posterior columns is also a controversial element of the presented research. The authors of the study are aware that, apart from sudden injuries resulting from fracture of the appendages, it is difficult to find an analogy to the model used. It is generally accepted that the intervertebral joints’ degeneration precedes IVD degeneration with segmental instability and height loss. And since such changes cannot be brought about in an animal model clearly and straightforwardly, they can be simulated by removing them.

## Conclusion

The presented research is an innovative method of describing the effect of complex fatigue loading conditions and the effect of support on articular processes on the formation of pathological changes within the lumbar IVD. Simultaneously, it has been demonstrated that the resulting structural changes significantly affect the mechanical properties of the outer layers of the AF. Changes in the height of the IVD are not a sufficient parameter to confirm the assumption that just lowering the height can influence the formation of pathological changes in the internal structure of the IVD. Analysis of the mechanical parameters of the AF’s outer lamellae has shown that long-term cyclic loading significantly affects the obtained Young’s modulus and stiffness coefficient.
